# Trends, Technologies, and Key Challenges in Smart and Connected Healthcare

**DOI:** 10.1109/ACCESS.2021.3079217

**Published:** 2021-05-11

**Authors:** Alramzana Nujum Navaz, Mohamed Adel Serhani, Hadeel T. El Kassabi, Nabeel Al-Qirim, Heba Ismail

**Affiliations:** 1 Department of Information Systems and SecurityCollege of Information TechnologyUnited Arab Emirates University 11239 Al Ain United Arab Emirates; 2 Department of Computer Science and Software EngineeringCollege of Information TechnologyUAE University 11239 Al Ain United Arab Emirates; 3 Department of Computer Science and Information Technology (CS-IT)College of EngineeringAbu Dhabi University 105947 Al Ain United Arab Emirates

**Keywords:** Artificial intelligence, big data, COVID-19, deep learning, healthcare, IoT, robotics, smart and connected healthcare

## Abstract

Cardio Vascular Diseases (CVD) is the leading cause of death globally and is increasing at an alarming rate, according to the American Heart Association’s Heart Attack and Stroke Statistics-2021. This increase has been further exacerbated because of the current coronavirus (COVID-19) pandemic, thereby increasing the pressure on existing healthcare resources. Smart and Connected Health (SCH) is a viable solution for the prevalent healthcare challenges. It can reshape the course of healthcare to be more strategic, preventive, and custom-designed, making it more effective with value-added services. This research endeavors to classify state-of-the-art SCH technologies via a thorough literature review and analysis to comprehensively define SCH features and identify the enabling technology-related challenges in SCH adoption. We also propose an architectural model that captures the technological aspect of the SCH solution, its environment, and its primary involved stakeholders. It serves as a reference model for SCH acceptance and implementation. We reflected the COVID-19 case study illustrating how some countries have tackled the pandemic differently in terms of leveraging the power of different SCH technologies, such as big data, cloud computing, Internet of Things, artificial intelligence, robotics, blockchain, and mobile applications. In combating the pandemic, SCH has been used efficiently at different stages such as disease diagnosis, virus detection, individual monitoring, tracking, controlling, and resource allocation. Furthermore, this review highlights the challenges to SCH acceptance, as well as the potential research directions for better patient-centric healthcare.

## I. Introduction

Smart and connected health (SCH) refers to the solutions or systems for digital healthcare that are fully connected and can operate remotely [1].The National Science Foundation (NSF) and National Institutes of Health (NIH) of the USA launched the “Smart and Connected Health SCH: Connecting Data, People and Systems” program in 2013 to accelerate the creation and integration of innovative information technology approaches [2]. Their vision was to develop the next generation of multidisciplinary research to enable existing and new scientific collaborations to work on innovative “smart” ideas.

Under the umbrella of smart cities, the use of digital technology, especially artificial intelligence, has been a focus of advancement, and healthcare delivery is no exception. Deloitte collectively identifies telecare, telehealth, telemedicine, m-Health, digital health, and e-Health services as connected health or technology-enabled care (TEC) [3]. Thus SCH is a new and promising interdisciplinary research field involving medical informatics, public health, big data, bioengineering, the telecommunication industry, and many other fields. SCH may be adapted to different critical health contexts, which may require resource-aware, time-constrained, complex, and secure healthcare transactions among various stockholders. SCH revolutionizes next-generation healthcare and has important potential benefits such as the acceleration of treatment and testing procedures, reduction in the cost of physician visits, efficient reactions to various emergencies and spread of pandemics, and improvement in the quality of patient care [4]. Furthermore, some emerging technologies have an immense capacity to transform certain areas of health and social care service. In addition, with an increase in the usage and popularity of smartphones and tablets worldwide without any age barriers, the prospects of mobile health applications have substantially increased in recent years in conjunction with numerous fitness and wellness applications. Other significant advances include the availability of “biosensing” wearables, such as automated blood pressure monitoring and glucose sensors, that allow real-time access to clinical data for patients and caregivers. Thus, using new digital technologies, SCH can empower patients and healthcare practitioners and help them enjoy better control of their own well-being, benefit easily from online knowledge, share views and concerns with their physicians, and find the right support and intervention.

The current COVID-19 global pandemic provides an excellent application scenario of the SCH: SCH adoption has proven its efficiency in pandemic management, tracking, and risk mitigation practices [5]. Never in modern history and on a global scale have we witnessed the emergence of such a breakout of a virus whose origins are largely unknown. COVID-19 was first reported in December 2019 in Wuhan from where it spread to different parts of China, ultimately becoming a global pandemic by March 2020 with a drastic shift from Asia to Europe and the USA. The pandemic has become an ordeal for humanity, with its spread causing fear, death, and havoc everywhere. Globally, as of 4:38 pm CEST, 5 May 2021 there have been 153,954,491 confirmed cases of COVID-19, including 3,221,052 deaths, as reported to the World Health Organization and a total of 1,170,942,729 vaccine doses had been administered as of 4 May 2021. Coupled with the existing financial crisis and rising global tensions, the situation is further aggravated by the extreme pressure on limited healthcare resources. Furthermore, many nations were forced to change the direction of economic development and modify their existing strategies, re-source utilization, and priorities to tackle and resolve the problems caused by this pandemic.

Unfortunately, cities have become hubs of rapid transmission in this pandemic. Rapid urbanization, population explosion, and global travel have worsened the situation. However, some countries were more resilient to the pandemic owing to the increased use of smart technologies such as the Internet of things (IoT), big data, AI, mobile technologies, and drones. In particular, China and Western democracies (the United States of America (USA), the United Kingdom (UK), Italy, Germany, Belgium, the Netherlands, France, and Spain), who were heavily affected in the initial three months of Covid-19 are known for adopting smart-city-based technologies [6]. Some examples of the results of the application of such technologies are listed here [7].

• The increased use of mobile technologies and smart devices has improved healthcare.
• More innovative SCH for monitoring individuals’ health can further improve healthcare.
• IoT integrates medical devices with the internet and provides/updates the real-time health status of patients to the consulting doctors.
• Allowing different healthcare providers easy access to repositories such as the national health databank has enabled better monitoring of the COVID pandemic [7].

Different governance mechanisms have been implemented by governments worldwide to control the pandemic, and surprisingly, their decisions were technology-driven and impractical at large. Although the extensive use of emerging technologies has improved the responsiveness to and control of the spread of the disease, this effect solely depends on the local governance and socioeconomic and cultural context [8]. Kummitha [6] compared two opposing approaches in the smart city framework, where China adopted a technology-driven approach (top-down, enforcing technology), whereas Western governments have adopted a human-driven approach (bottom-up) to control the transmission of COVID-19. The findings in the first case highlight that although the technology-driven approach may be more productive in terms of identifying, isolating, and quarantining infected individuals, it also suppressed and censored the citizens. Furthermore, human interaction with technology is mediated by the political and institutional context in which the technologies are implemented. China employed its well-established surveillance system and placed cities under complete quarantine using a proactive strategy. Scientists and engineers in most developed nations were fiercely competing to develop a vaccine, to set-up more testing facilities, and to enhance monitoring systems [7]. With vaccines now available globally, some people are becoming less concerned with mask wearing and social distancing, despite the fact that the majority are still not vaccinated.

This research work delivers a comprehensive state-of-the-art review on SCH systems, which assists the community of researchers and other stakeholders in the healthcare field to compare, comprehend, and build a clear understanding of the benefits of SCH systems. The proposed architecture is considered as a reference model to support healthcare stakeholders in realizing the technological perspectives, integrating the different SCH components, and building a complete SCH system. It emphasizes as well the main challenges which face the adoption of these systems in terms of usability, accessibility, data management and analytics, monitoring, and mobility handling. Additionally, it provides a future vision of the next-generation SCH solutions and systems.

This research aims to answer the following research question: how can we develop effective intelligent SCH systems and strategies to provide smart, efficient, proactive, patient-centric healthcare? To answer this question, we propose the following contributions.

• Provide a classification model and literature review on various existing SCH models, solutions, and architectures.
• Propose an architectural model that captures the SCH solution’s technical aspects, its environment, and the key stakeholders involved. Furthermore, the proposed model incorporates core innovations such as IoT, Big Data analytics, and AI to assist the primary stakeholders in improving the efficiency of the healthcare system.
• Layout a case study on the adoption of SCH supported by evidenced data to combat the COVID-19 pandemic and illustrate how the pandemic was differently handled in terms of leveraging the power of various SCH technologies to identify, monitor/track and control the disease/virus; additionally, the present research focuses on the optimal technical resource allocation to combat the pandemic.
• Identify key SCH challenges, and offer prospective research and technology paths for next-generation SCH, as well as provide recommendations for future SCH system implementations, taking into account the lessons learned from the present pandemic.

The rest of this paper is organized as follows: Section 2 presents the methods used for our scoping review and Section 3 provides the results with a comprehensive literature review, classification, and comparison of the SCH solutions. Section 4 introduces SCH solutions and their related enabling technologies and proposes an SCH architecture. Section 5 presents a case study analysis on SCH adoption for managing and combating the COVID-19 pandemic. Section 6 identifies and explains the main challenges to the SCH solutions and discusses some solutions to these challenges and identifies some research directions towards the future generation of SCH. Finally, the last section concludes the study and indicates some research forecasts for upcoming years in the field of SCH.

## II. Research Strategy and Study Selection

To classify and process the existing literature that reports the implementation, architecture, and solutions of SCH in healthcare, this study used the scoping research review approach.

### A. Search Strategy

Papers were sought from scientific research databases and search engines (e.g., Scopus, IEEE Xplore, PubMed, ScienceDirect, and Publons) using specific keywords. Besides, we considered few relevant white papers. The following are examples of the keywords we employed: smart and connected health, AI in SCH, blockchain in SCH, IoT in SCH, robotics in health, applications of SCH, and SCH frameworks.

### B. Study Selection

**FIGURE 1.**
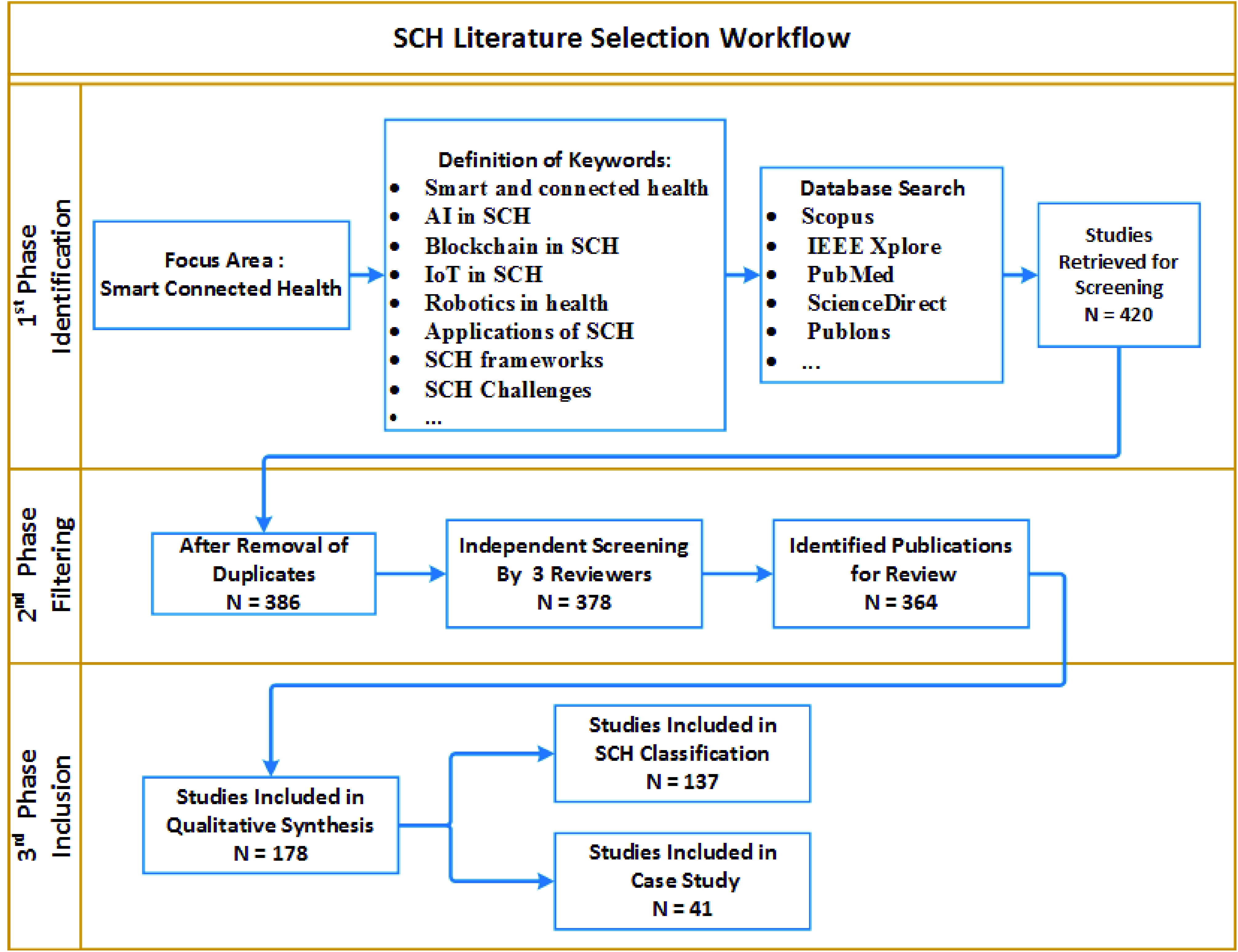
Literature selection workflow.

The study selection procedures were followed after papers were selected based on the stated keywords. The papers collected have been screened and duplicates have been excluded with their digital object identifier (DOI). Initially, we divided the research papers into several groups according to the keywords used. This process was iteratively applied by all our research team members to ensure the validity of the conclusions. Consultations were made with clinicians and specialists in the medical field to further confirm the developed model. In three phases, the technique used for the review of the literature was carried out. Fig. 1, summarizes the phases introduced for the search, the selection of literature for SCH analysis, and the outcomes.

### C. Scoping Review Results

**FIGURE 2.**
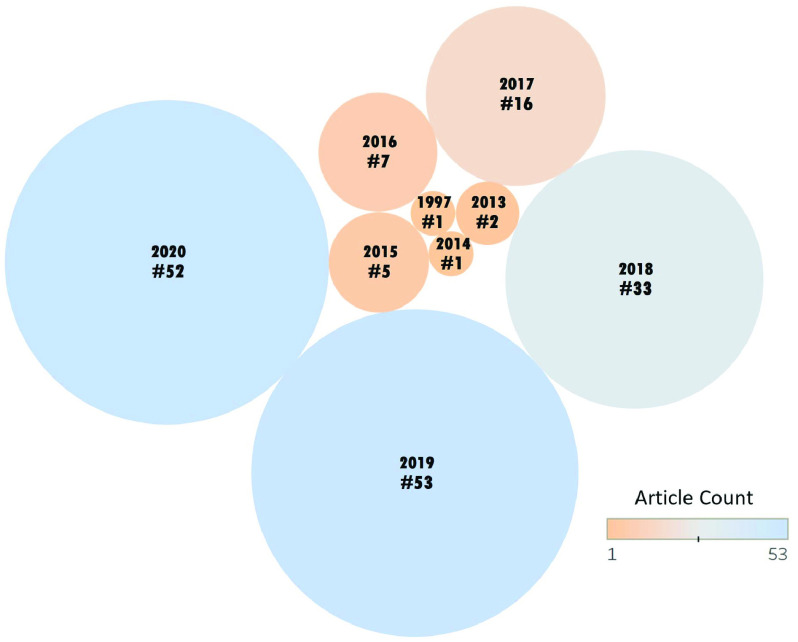
Articles by year of publication.

We reviewed over 360 research papers and categorized the studies into five representative areas. Interesting observations concerning publishing patterns were found by the quantitative study of the article meta-data. Selected articles with respect to the year of publication and the count in the respective years are represented by a bubble chart in Fig. 2. An increasing trend in the number of collected articles can be noticed from 2015 to 2020. The meta-data study also reveals that research on SCH based articles is published in a broad range of journals on sensors and technologies such as AI and IoT in healthcare. Sensors, which publishes papers based on technological solutions and their applications and impacts, is the top journal by the number of articles (16) followed by IEEE Access /Conferences/Transactions (13), IEEE Journals (6), Journal of Medical Internet Research (5), Scientific Reports(4), Artificial Intelligence in Medicine (3), Future Generation Computer Systems (3), Journal of Medical Systems(3), Journal of Ambient Intelligence and Humanized Computing (2), and International Journal of Advanced Computer Science and Applications(2). Overall, there appeared to be a balanced mix of publications that concentrated on SCH technologies and applications in healthcare.

## III. Smart and Connected Health: Review, Classification, and Comparison

In this section we further review the literature and proposes a classification and a comparison of the existing work related to SCH, based on the enabling technologies, disease and pandemic monitoring (the application context), and futuristic systems.

**FIGURE 3.**
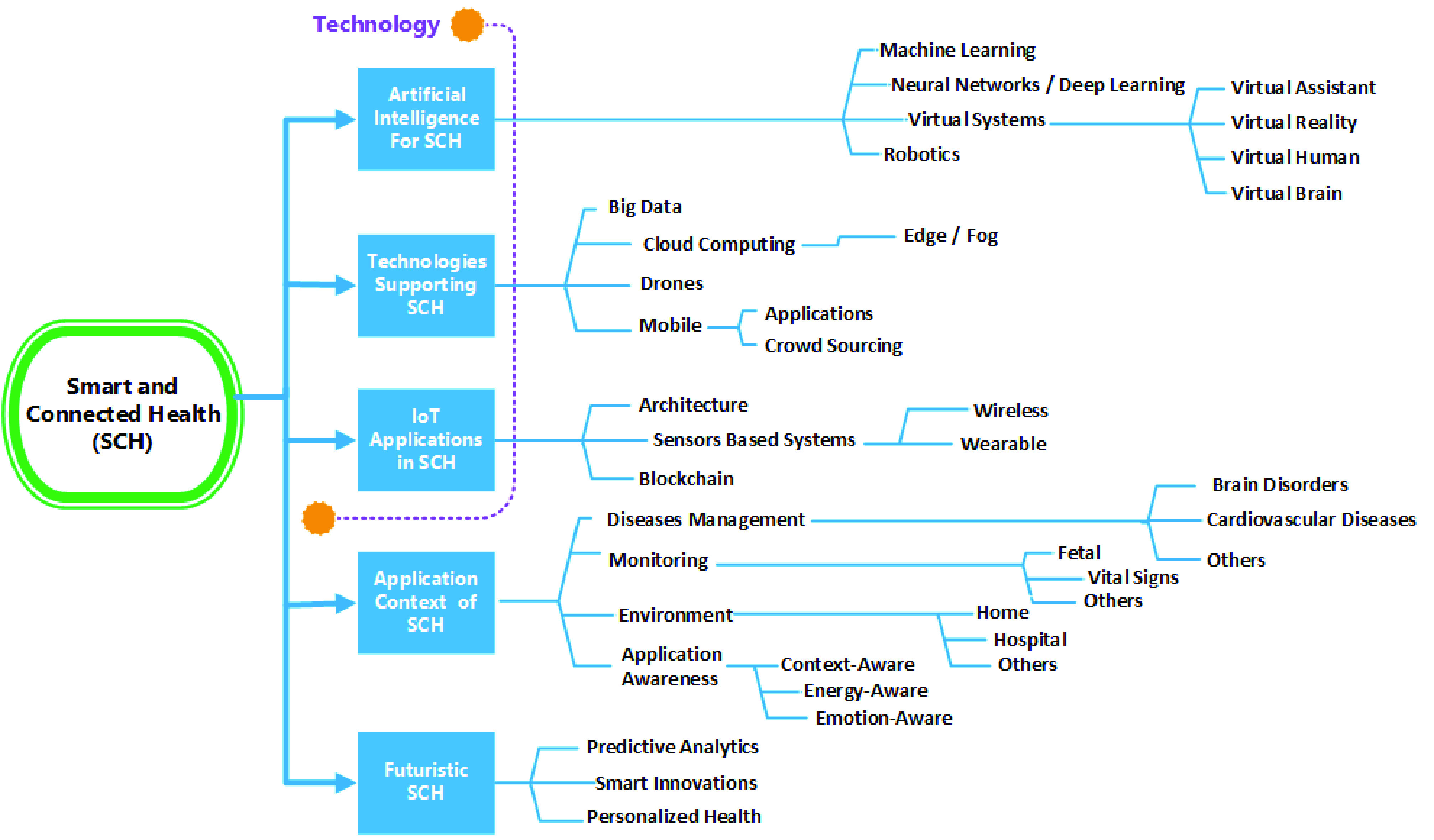
Classification of smart and connected health.

The classification shown in Fig. 3 has been derived from a scoping review of the literature on state-of-the-art SCH. IoT has shaped healthcare governance, and indeed, SCH in conjunction with smart devices and AI allows healthcare providers to offer more tailored and personalized healthcare solutions. Thus the first three clusters were based on studying the current trends, advancements, and implementations of SCH coupled with AI, IoT, and other supporting technologies. In addition, further clusters were introduced to focus on the application context of SCH and futuristic works on SCH.

In the subsequent sections, we present and evaluate each of the above-mentioned clusters and summarize the work performed within each cluster.

### A. Artificial Intelligence for SCH

This cluster explores the healthcare impacts of AI and its various branches namely machine learning, deep learning, robotics, and virtual systems through a scoping literature review as seen in Table 1. Commonalities occur in a few of these settings, and the differences are also apparent.

**TABLE 1:** Studies on AI-Enabled SCH

	AI-Enabled Smart and Connected Health						
	Machine Learning	Neural Networks / Deep Learning	Robotics	Virtual Systems			
				Virtual Assistant	Virtual Reality	Virtual Human	Virtual Brain
Referenced Papers	[9], [10], [23]	[11], [12], [24], [25], [34], [35]	[13], [14], [15], [26], [27], [28], [36],, [37], [38], [39], [40], [41], [42], [43], [44], [45], [46], [47], [48], [49], [50]	[16], [17], [29]	[18], [19], [30], [31]	[20], [17], [32], [33]	[21], [22]

AI can be applied to structured and unstructured healthcare data. Common AI techniques include machine learning (ML), such as the classical support vector and neural network, and modern deep-learning based on neural networks. ML techniques typically analyze structured data such as data from images, genetics, and electrophysiological data. The ML methods used in medical applications attempt to cluster the characteristics of patients or infer the probability of disease outcomes [51]. For example, statistical deep learning-based pattern recognition techniques proved good results when used for endocardium tracking in ultrasound data. Additionally, deep learning and AI techniques proved good potential for processing cardiac MRI. Furthermore, Convolutional Neural Network (CNN) gave high accuracy results when calculating coronary artery calcium in cardiac CT angiography using the supervised type of ML [51].

Gaussian process regression (GPR) along with the relief feature selection algorithm was developed to achieve better performance than other ML algorithms in estimating systolic blood pressure and diastolic blood pressure BP [9]. In another study [10], a diabetes prediction system using ML data mining techniques were proposed. That system employed an ensemble decision tree algorithm for feature selection. The following three layers constitute a deep neural network: input layers with treated and untreated data; hidden layers where the strength of the connection is trained as a weight; and output layers of trained results. Controlling the weight of training data in deep neural network layers enables repetitive learning. An ambient context-based model developed using deep neural networks for health risk assessment is proposed in [11].

Robotics is currently employed in various applications, primarily in challenging environments, ranging from material handling in labs to surgical procedures, and patient care [47]. Multi-agent intelligent healthcare systems that can enable robots to select the most useful plan for unmanaged situations and communicate the choice to the physician for approval have been proposed in [45]. Home robots that support elderly people and help in coping with the problems of aging, such as disorders affecting memory, motor functions, and muscle weakness, can overcome the shortage of caregivers and healthcare providers [14], [45], [53]. Robotic-assisted minimally invasive surgeries such as heart surgery [46], neurosurgery [44], liver surgery [42], and ultrasound [28] and MRI-guided [39] robotic interventions are more established now. In addition to improving surgical workflow, robotic-assisted surgical interventions have facilitated precise robotic bone machining, enhanced bone implantation, optimization of implant positioning and alignment, and reduced running time. Surgical maps are preoperatively generated by AI-based systems to achieve optimum precision [48]; thus, before initiating a procedure, the surgeon can review and modify the surgical plan and conduct a mental walk-through of the procedure.

Voice-activated assistants have numerous potential healthcare use cases, including those in the fields of education, health tracking, and monitoring; further, they can provide assistance in locating healthcare providers. The types of health and fitness applications available for voice-activated assistants fall in the categories of health education, fitness and training, nutrition, brain games, health surveillance, motivation, and meditation [16]. The advanced smart cloud-based emergency aid chatbot named SPeCECA [29] assists victims or accident witnesses in preventing the worsening of the victims’ condition and preserves physical integrity before physical assistance arrives, thereby significantly improving the likelihood of the victim’s survival. This solution is a smart, connected smartphone application that acts as a virtual assistant and provides an online human-bot interface to support individuals in an emergency.

Virtual reality (VR) and augmented reality (AR) are novel technologies that can create an artificial world and allow the user to immerse in and interact with it; the use of these technologies can certainly raise the standards of healthcare and medicine [18]. These technologies include devices such as head-mounted screens, and haptic devices together with AR glasses, software, and tracking sensors. VR is extensively used in plastic surgery and can be potentially used in the areas of any surgical preparation, navigation, and training [19]. VR-based virtual training has been comprehensively studied. Several commercial VR products are already available in medicine for educational purposes.

Virtual humans [20], i.e., embodied conversational agents (ECAs) have been employed to conduct clinical face-to-face interviews wherein each patient answers interview questions and a decision-making algorithm identifies the participants with and without major depressive disorders (MDD) according to DSM-5 criteria. ONParkinson [32] is another interactive conversational agent aimed at offering intelligent resources to strengthen people with Parkinson’s disorders.

The Virtual Brain [21] is a neuroinformatics framework for complete brain network simulations for the replication of brain dynamics at the macroscopic level of the brain organization, allowing rapid data analysis and results in visualization. Virtual neurosurgery, mimicking the actual surgery of brain tumor patients by modeling the brain network and simulating neural activity has been explored [22], unlocking interesting research directions in the field of medicine.

### B. Technologies Supporting SCH

This section explores the data and processing technologies and platforms supporting SCH, and the related studies are shown in Table 2.

**TABLE 2:** Technologies Supporting SCH: Referenced Studies

	Technologies Supporting SCH					
	Big Data	Cloud Computing		Drones	Mobile Computing	
		Fog	Edge		Mobile Apps	Mobile Crowdsourcing
Referenced Papers	[45] [52], [53], [65], [66], [70], [71], [72]	[54], [55]	[56]	[57], [58], [68], [69]	[59], [25], [60]	[61], [62], [63], [64]

The healthcare sector has become an emerging hub of big data users with the advancement of medical sensors in our daily life. For instance, the research kits from Fitbit and Apple can provide researchers with access to vast stores of user biometric data, which can then be used to test hypotheses such as those on nutrition, fitness, disease progression, and treatment appropriateness. [52]. While general-purpose storage technologies, e.g., relational and NoSQL databases supported by horizontal scaling and data replication addresses the storage of big healthcare data relatively successfully, the processing and analysis of healthcare data are domain-specific tasks. Three challenging areas of big healthcare data analytics are the image processing of medical images, genomics analyzing genome-scale data, and signal processing of data generated by medical sensors. A conceptual distributed three-level data fusion model for smart healthcare is proposed in [45].

Care providers may need to make critical decisions about the health of their patients using these huge volumes of health information provided in a multitude of data formats. State-of-the-art healthcare systems with IoT and big data analytics are proposed in [53], [66], [67]. The key elements of big data analytics are the data elements, functional elements, human elements, and security elements [66]. Deep-learning techniques can be utilized for health data analytics using distributed and scalable architectures [67].

The convergence of the IoT and the cloud introduced the so-called cloudIoT model, which aims to broaden both technologies and realize the next generation of smart healthcare apps. An innovative and sustainable healthcare system based on the game theory approach for cloudIoT [73] for ambient assisted living environments, based on the game theory approach can improve the efficiency and latency of the network. In fact, with the exponential growth of sensor-generated data, several weaknesses have been observed in cloud-centric processing and analytics. The quality of the service is significantly affected by the speed of the Internet connection, which also raises the question of availability. In particular, where real-time processing is required, such as early disease detection or diagnosis, the optimal solution is to distribute the workload between cloud and fog nodes, often allocating the model training to the cloud and decision-making to the edge [54]. Partitioning and distributing data and processing is a promising trend, particularly for low-powered mobile devices.

Drones demonstrate tremendous potential for healthcare and medicinal transformation in the 21^st^ century [58]. The MEDIDRONE [57] was developed to provide inhabitants of rural villages with on-time emergency services and health insights. The services will include cost and time-effective transportation of vital drugs such as antivenom for snakebite and dog bite to avoid deaths arising from these causes. Drones may be used for rescuing victims and providing food, water, and medicines in disaster-relief operations. In addition, they enable life-saving actions such as rapid organ transfer between hospitals by bypassing crowded traffic [69].

Trending mobile health (mHealth) relationships with big data and insights can efficiently fulfill national healthcare demands in less-developed countries without fully open healthcare systems [74]. Nowadays, a wide range of mobile devices of various sizes, from cell phones and smartwatches to small phones and tablets, along with integrated wearables, implants, and location-based trackers and sensors are available. A smartphone can be used by a physician as a fully unified method to diagnose and treat patients in a variety of ways: for example, it can be used to access medical records; it can be used as a stethoscope; the camera can record while offering a magnified view of hard-to-reach areas, or it can be connected to an endoscope [75]. An Android mobile-based early warning system proposed in [76], comprising two sections, was introduced in an attempt to decrease the recovery time within the first hour following a traffic incident. The front-end patient application allows sending messages conveying the type of incident to emergency services, whereas the back-end at the emergency side receives the notifications and transmits a warning message to the suitable specialist doctor. A ground-breaking approach to health-state tracking using the smartphone application “Neural Impairment Test Suite” (NITS), which is available on Google Play, is specifically tailored for subjects suffering from neurodegenerative conditions and intended to regularly monitor their state of health [77]. This program was implemented based on medical information gathered a priori and summarized following the SAGE methodology (self-administered cognitive testing). In mobile application development, user-centered design and intelligent learning systems are the primary concerns for delivering flexible, personalized, and quality treatment for each patient [78]. Mobile apps are employed in various situations such as for the enhancement of home health [79], accident detection and smart rescuing [41], patient education [80], fetal monitoring [81], smoking cessation [25], asthma management [78], and for monitoring hypoglycemia [82] and eating behaviors [60].

An emerging technology called mobile crowdsensing (MCS) has grown with the massive deployment of smart devices, and with the ubiquity of the Internet, appropriate data can be accessed for proper diagnosis and treatment in healthcare MCS (HMCS) [61]. However, this technology utilizes the confidential health details of patients, and any unwanted disclosure may have serious implications for the well-being of the patient; thus, privacy and data protection are major concerns in HMCS.

### C. IoT Applications in SCH

The fourth industrial revolution in recent years has contributed to the so-called growth of the Internet of Robotic Things (IoRT) [26], integrating robotics, cloud computing, and IoT. This offers several advantages in providing diagnosis and health information in real-time, intending to reduce the risk of human errors. The IoT has been commonly accepted as the dominant technology in SCH for alleviating the stresses on healthcare systems, and hence, it has been widely researched [83]. Major IoT e-health benefits are cost reduction, interoperability, ease of use, real-time analytics, availability, and lifetime monitoring [84]. The studies on IoT applications in SCH can be categorized as those on IoT architecture/framework, sensor-based systems, and IoT applications in blockchain (see Table 3).

**TABLE 3:** IoT Applications in SCH: Referenced Studies

	IoT Applications in SCH			
	Architecture / Framework	Sensor-based Systems		Blockchain
		Wireless	Wearable	
Referenced Papers	[85], [84], [99], [100], [101], [104], [105], [106], [107], [108]	[86], [87], [102]	[88], [89], [103]	[90], [91], [92], [93], [94], [95], [96], [97], [98]

The majority of studies in the literature have emphasized the use of sensors to monitor patient health [79], [80], [132], [134], [135]. Networked sensors, either embedded in our living environment or worn on the body, allow us to gather extensive knowledge about our physical and mental health. Combined with the new generation of smart-processing algorithms, the accessibility of information at large scales and spatial longitudes will accelerate development in the medical sector [109]. Implementation of all critical sensors as lightweight, compact, and externally wearable nodes is preferable. A knee injury rehabilitation device, built using wearable accelerometer sensors on both sides of the knee to measure the knee’s position and angle, is an implementation of the proposed model [110]. The SmartPANTS [111] system was specifically conceived as a smart home rehabilitation platform for patients recovering from a brain stroke. It includes several wearable devices and has a software interface that provides real-time feedback. Wearable health-monitoring devices using three body sensors and exploratory data analysis were developed in [112] to support real-time monitoring of patient health.

IoT-aware architecture for Smart Hospital Systems (SHS), which was proposed in [85], can monitor all environmental factors and physiological parameters of patients in real-time and transmit them to a control center. This architecture has three key parts: (1) the Radio-frequency identification (RFID) enhanced wireless sensor network (WSN), called hybrid sensing network HSN; (2) the IoT smart gateway, and (3) data visualization and management user interfaces. The reliability block diagram (RBD) is recommended in [107] to set up a framework for reliability modeling and studying end-to-end IoT systems. iResponse [108] is a five-module technology-driven framework for autonomous pandemic management that includes policy implementation, resource preparation and provisioning, data-driven planning and decision-making.

Biometric data are the key to IoT operations, and biometric characteristics are used as signatures or as identity standards because they cannot be borrowed, purchased, or lost and are difficult to replicate or duplicate. Smart healthcare and IoT has ushered in a new paradigm in biometric data applications [113]. A bridge between a sensor network and the Internet exists in most of the IoT-based patient monitoring systems, particularly at smart homes or hospitals. In [114], the emphasis is on the advantage of the strategic location of these gateways to deliver many higher-level services such as local storage, local real-time data processing, and embedded data mining to alleviate the concerns of energy efficiency, scalability, interoperability, and reliability.

Security is one of the most critical issues of any type of information system. IoT technology constitutes an information network that enables data exchange between vast numbers of users and devices, and hence, security is a requirement. Two-factor authentication, with a smart-card and password, offering high security with the minimal computational cost is suggested in [115]. To mitigate user spoofing in smartphones, a system that uses accelerometers embedded within smartphones to exploit the correlations inherited from a user’s walking traces and extract the gait patterns is employed in [116]. A potential Internet model called Named Data Networking (NDN) was recently proposed to strengthen and simplify these IoT connectivity issues. NDN allows users to collect data by names, regardless of the hosting entity in question. In [117], [118], the basic features of NDN architecture were leveraged to develop and verify an NDN-based smart health IoT system. Blockchain, which is based on P2P (peer to peer) network computers (nodes) in the healthcare system, can be used to protect the management and analysis of big data in healthcare [95]. This would increase the security of the Internet of Things (IoT) and ensure the secure connectivity of medical equipment’s. However, blockchain is computationally expensive and demands high bandwidth and extra computational power. Few works [91], [94] have attempted to resolve the challenges of resource-constrained IoT devices using IoT devices with blockchain. Currently, some geospatially enabled blockchain solutions [93] that use a cryptospatial coordinate system are available to add an immutable spatial context that the standard blockchains lack.

### D. Application Context of SCH

The studies referencing application context in SCH are classified into four main categories: disease management, monitoring, environment, and application awareness as depicted in Table 4).

**TABLE 4:** SCH Application Context: Referenced Studies

	Application Context of SCH			
	Disease Management	Monitoring	Environment	Application Awareness
Referenced Papers	[119], [120], [78], [89], [125], [126], [134], [20], [135], [10], [82], [130], [19], [89], [77], [127], [87], [25], [15], [9], [122]	[121], [122], [95], [127], [128], [129], [18], [88], [106], [81], [140], [60], [103], [143], [144]	[13], [111], [123], [80], [31], [79], [76], [130], [127], [136], [137], [41], [141], [30], [142], [29], [129], [82], [119], [111], [122], [16], [135], [130], [121], [89], [134], [135], [128],	[56], [11], [124] [131], [132], [133], [138], [139], [71]

SCH plays a major role in the field of medicine, from remote diagnosis to complex teleinterventions. It has also evolved to fulfill the growing demands for self-monitoring and disease management. Medtronic [145] is working with Canary Health [146], a digital wellness technology provider, to provide its mobile chronic disease management services. The CDC-recognized Diabetes Prevention Program was developed to improve habits with structured lifestyle-change programs for people with prediabetes [52]. A medical diagnostic tool that enables early diagnosis for brain stroke patients using a wearable electromagnetic head imaging device is proposed in [89]. Intracranial hematoma (ICH) is the leading cause of permanent disability, and every second, millions of brain cells die from the onset of ICH in a patient. The guiding factor for the timely treatment of the same is the prompt diagnosis supported by a portable multislice microwave imaging system [130].

Health monitoring relies on rigid electronic housing coupled with aggressive adhesives and conductive gels, which cause discomfort and damage to the skin. In [122], all-in-one, portable, and stretchable hybrid electronics with integrated long-range communication are presented. Real-time analysis of the values of the biomedical electroencephalogram (EEG) signals and automated detection of epileptic seizures before onset can alert patients; consequently, protective measures can be adopted [12]. Potential cardiac pathologies [128], based on data obtained from a 24-h Holter recording can statistically evaluate the heartbeat sequence linked to each patient, and further, these markers can be used as inputs for a neural network with multilayer feed-forwards. Health monitoring ranges from fetal heart rate monitoring [81], which tracks the well-being in utero, to elderly monitoring [18], [129] of people suffering from a moderate cognitive disability such as Parkinson’s or Alzheimer’s disease.

The applicability of SCH is suited to various environments, including, smart homes, health index monitoring [123], and smart home rehabilitation networks [111] for patients suffering from brain injuries; in these cases, smartness is incorporated using IoT systems. Training is another notable SCH-enabled environment, wherein medical education can be delivered and enhanced via smartphones [79], [80] and augmented-reality-enabled telemedicine platforms [31]. Disaster emergency management with augmented reality using a triage algorithm with smart glass applications is simulated in [136].

Context computing is an ambient intelligence research branch that has rapidly evolved with intelligent smart health application solutions. Ambient context-based modeling was reported in [11]; therein, a deep neural network was used for health risk assessment based on evidence from individual health problems subject to the environmental context. Context-aware solutions for multimodal data compression, in-network processing, and edge-event detection in multi-edge cloud-based systems [56] are promising. A context-aware and self-adaptive model serving as an IoT security management platform that can autonomously track, interpret, and respond to a host of security contexts are proposed in [124]. That model harnesses the benefits of fog computing and provides flexibility and open networking to handle any smart device.

Optimizing the energy consumption of IoT devices in continuous data-flow applications is a challenging problem highlighted in [132], [138]. Anomalous nodes in the network will increase energy consumption. To optimize the effectiveness of continuous data flow applications, a mathematical programming approach such as a lightweight method of anomaly detection to assess node reliability [147] can be adopted. An energy-efficient hardware prototype developed on an IoT-based microcontroller platform was presented in [132]; this prototype can compress the real-time electrocardiogram (ECG) signal through sparse time-frequency domain encoding. An energy-aware cyber-physical therapy system (T-CPS) framework [133] that used multimodal sensing to provide energy-efficient affordable therapeutic services has been reported.

### E. Futuristic SCH

The future of SCH will definitely rely on smart hospitals and smart patient-physician interactions using smarter and innovative solutions. In the case of accidents, mobile apps will allow the network to geographically connect to the nearest hospital and transfer data before admission [76], thereby possibly saving lives. Adoption of digitally enriched technologies that facilitate novel self-care mechanisms is expected in the future. Consider, for example, flash or continuous glucose-monitoring systems in which the sensors can act as display devices, can provide hypoglycemic and hyperglycemic warnings, and wirelessly transfer glucose data to a reader or mobile application. Eye-tracking can be used to guide the patient-centered product development of novel drug delivery systems (self-injection systems) [125] through the approach of usability testing. This development can substantially improve the interplay between the constituent parts (including sensors, infusion pumps, remote control, and mobile apps) of the linked system, thus improving the overall product efficiency.

Predictive analytics plays an important role in the development of decision-support systems for the precise prediction and diagnosis of diseases. Its advantages in the healthcare sector far outweigh the potential challenges [148]. A novel decision-making model proposed in [149] was based on the IoT method to diagnose and control type-2 diabetes; this model was implemented using WBAN and a mobile device interface. The Allgemeines Krankenhaus Informations Management project was initiated [150] at Vienna General Hospital several years ago and since its ability to support standards such as Arden Syntax and the integration of clinical decision support systems into clinical routine were demonstrated, the number of clinicians in favor of decision support has substantially increased. Table 5 shows a few of the referenced studies in futuristic aspects of SCH.

**TABLE 5:** Futuristic SCH: Referenced Studies

	Futuristic SCH		
	Predictive Analytics	Smart Innovations	Personalized Health
Referenced Papers	[155], [100], [101], [150], [151]	[86], [152], [153], [154]	[67], [155]

PillSense proposed in [103] is a smart, non-invasive, mobile, and energy-efficient system that utilizes a pill bottle equipped with various sensors for detecting the opening/closing of the pill bottle, movement tracking, and weight medication measurement. Smart personal protective equipment (PPE) proposed in [153] embedded with sensor technology is used to monitor the health of workforces and check their proximity to harmful chemicals and hazardous areas. A smart mirror developed in [155] provides health indicators such as body temperature and heart rate in addition to enabling the observation of changes in skin characteristics such as color, texture, moles, and rashes as the monitored person performs his/her daily routines. All of these parameters reflect significant health markers, and this concept is the core principle of smart mirror technologies where at-home surveillance offers new possibilities for more reliable and practical diagnostic measurements. The smART+ feeding tube is a part of the smART+ Platform reported in [160]; the tube is fitted with multichannel bioimpedance sensors to identify both minor and massive reflux events, stop aspiration, and avoid feeding and inflating an esophageal balloon when a reflux event occurs. The platform also provides continuous and real-time tracking of urine flows to issue low-urine warnings. Advanced Deep Learning techniques can definitely help build smart context-aware disease diagnosis, analysis, and recommendation solutions for predictive treatment [67]. Recommendations for human interpretable care decisions will provide care workers with the means to incorporate appropriate care and treatment plans.

Using 5G and AI-based technology, we can drastically increase the level of patient care in rural areas and expand medical facilities in hospitals. 5G technology offers optimal alternatives, beyond wireless technology, to ICT-assisted health systems. A 5G-enabled ambulance can provide remote diagnosis and services, can automatically respond to an emergency call, and can choose an optimal route that can prove lifesaving. The high transmitting speed of 5G and its ultra-reliable low latency communication (URLLC) enables the remote operation of robots [156]. Network stability is a major problem in the previous generations of ICT-assisted remote surgery systems. Disconnection of a moment or even a poor level of communication will result in operation failure and may cause the death of the patient. This can be resolved via 5G technology that realizes the recreation of a three-dimensional (3D) image and relays streaming media in high definition. S-MAPLE developed in [157] is a next-generation portable smart health record management system with secure near-field communication (NFC)-enabled mobile devices that offer safe and convenient access to up-to-date health records and facilitates hospital mobility for patients. An accredited medical practitioner can directly and selectively access it with their mobile devices through NFC wireless interfaces.

The autonomous ventilator proposed in [158] is a system that can continually track the patient’s response to ventilation while changing ventilator settings to conveniently provide the patient with optimally administered oxygen; this is a promising system. In the absence of professionals in respiratory care, the automated ventilation proposed in [159] with AI innovations adds significant value to remote environments.

## IV. Smart and Connected Health, Solutions, and Enabling Technologies

We discuss SCH solutions and the technologies that support them in this section, as well as propose an SCH architecture and detail an implementation scenario of CVD monitoring and how components and technologies included in the architecture can be used in practice.

Data constitute the core of SCH wherein different technologies are used to gather, process, interpret and visualize data for more effective data-driven decision-making. The real challenge is not providing data but interpreting it to obtain valuable insights. Hospital reports contain historical data on patients and their medical records. Such data can be analyzed to detect correlations, patterns, and trends that can predict the probability of any disease occurrence in patients. Further, such an analysis can help select the best treatment options for the patients. For example, advances in automated image processing in radiology and pathology have revealed interesting insights that have improved the process of diagnosis. Machine learning is more effective in detecting lung cancer compared with detection by humans. Surgical data science is an evolving area in medicine and is witnessing fierce competition among medical equipment developers in terms of using approaches such as data mining to better compete in the market. Compared with humans, deep-learning algorithms can better detect trends when analyzing the patient’s historical data [13]. All the above technologies and others are leveraged to build an efficient and data-driven SCH architectural solution that will serve extensive needs from disease monitoring to pandemic tracking and isolation.

### A. SCH Architecture

In this section, we propose an SCH architecture that we believe captures the technological aspect of the pandemic’s solution, the environment in which this solution can be applied, and the primary involved stakeholders. The proposed SCH architecture may be used according to the particular application for which it is used. Therefore, its adoption is subject to the context of the application, its purpose, and the main beneficiary of such solutions.

**FIGURE 4.**
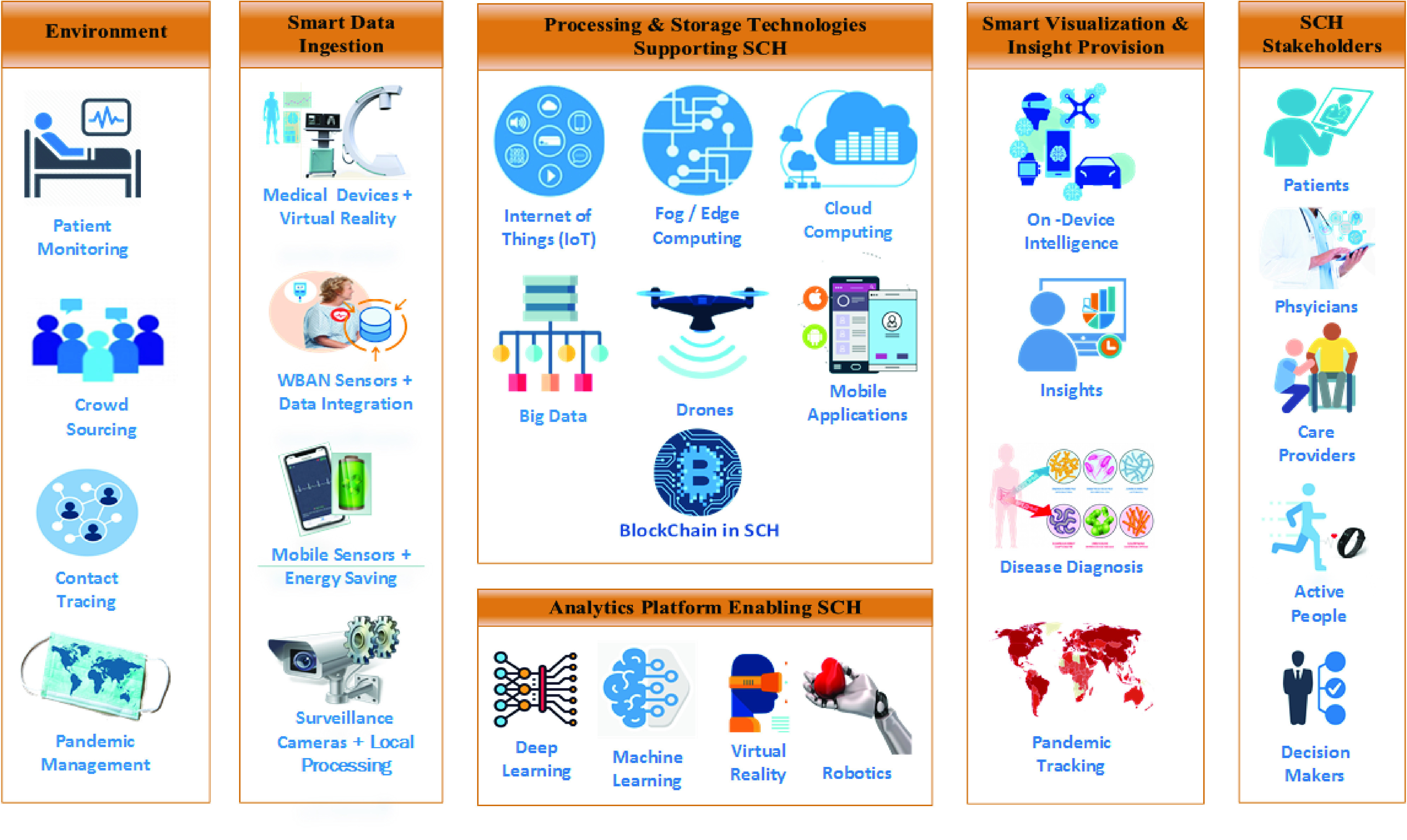
Building blocks of smart and connected health (SCH) architecture.

As shown in Fig. 4, once the environment is established, the process follows the big data value chain approach: data ingestion, preprocessing, processing and storage, analytics, and finally, visualization and insight provision. The uniqueness of this SCH architecture is signified by the use of smart solutions at different stages as explained in subsequent sections.

#### 1) Environment

From the perspective of IoT, the SCH system resembles a single recognizable medical “thing” linked to the Internet (IoT). On a large scale, the SCH system may involve several “medical things” that can be interconnected through the Internet. For example, the management of patient data involves different settings for use in hospitals, homes, and ambulances; at times, this management is performed through smart remotely applied technology. Exploring new approaches to automating large-scale data collection in such varied scenarios is important. Crowdsourcing [141] is one viable strategy as it can effectively utilize the online collective knowledge of the community. Epidemics caused by diseases such as COVID-19 and SARS spread to different continents and affected both animals and humans. To tackle such pandemics, SCH can be efficiently employed in contact tracing and pandemic management.

#### 2) Smart Data Collection

The data ingestion layer represents the founding stage of the data-driven architecture presented here in Fig. 4. This layer emphasizes smartness in the data ingestion process: e.g., the integration of augmented-reality-enabled medical devices that may help surgeons schedule their operations and prepare them for any unexpected situations. A wireless body area network (WBAN) connects different healthcare providers and related stakeholders over the internet [148] and allows sensory data to be collected and transmitted to the cloud in real-time. IoT surveillance cameras and mobile devices are typically energy-consuming solutions; thus, they require components that are “smart” in terms of energy supply and storage efficiency in addition to embedded data collection repositories, processing, data compression and transfer optimization algorithms [160].

#### 3) Processing and Storage Technologies Supporting SCH

Application-specific processing, including local data collection, preprocessing, transmission, and retrieval over fog and cloud platforms are the essence of this layer. Mobile applications and drones that require real-time processing in tasks such as capturing violations of face-mask usage and implementing deep-learning techniques are some examples of such processing. AI seamlessly integrates virtual clouds and functional blockchain applications to assure secure health data transactions. Another example of smart processing is the use of a smart decision support model (SDSM) [160] incorporating a resource meter that measures a series of metrics to determine the availability of resources (e.g., memory, disk, and battery) for mobile devices and rapidly determines where each process or subprocess should be executed—online (on the mobile device) or offline (transfer to a backend server).

#### 4) Analytics Platforms Enabling SCH

Predictive analytics can be a central feature in healthcare delivery, and such platforms primarily rely on AI, deep learning, virtual reality (VR), and robotics. Analytics derives value from health data; thus, advanced analytical models are important for data-driven optimization and real-time decision-making in a health emergency [66].

#### 5) Smart Visualization and Insight Provisioning

Real-time monitoring and alerting in case of seizures and drowsiness during driving, when blood sugar levels drop, or in fall detection among elderly people are different scenarios wherein smart technologies are prevalent. With more connected health devices, pandemic diagnosis, tracking, and visualization of data are critical for decision-makers. For example, smartness in visualization can be achieved by customizing the outputs according to the device screen size or resource availability [161].

#### 6) SCH Stakeholders

Successful SCH implementation will help healthcare planners in improving organizational and decision-making processes. SCH stakeholders, namely physicians, patients, healthcare providers, and active people, can have different roles and perspectives in the system depending on the adopted architecture.

### B. SCH Architecture Application Scenario

The leading cause of death worldwide is cardiovascular disease (CVD), and electrocardiography (ECG) is a commonly adopted method for quantifying cardiac activity to detect any heart defects [162]. A typical SCH application scenario is depicted in the following example. Consider a patient being monitored for heart diseases in a hospital facility. The patient’s ECG data is collected using wired or wireless biosensors. In the case of home or ambulatory, data is relayed to a mobile device having smart data ingestion capabilities such as mobile resource-saving. Any abnormalities in the ECG signals such as fluctuations will be detected during processing which is performed at Fog/Edge/Cloud Computing environment. Through which, the ECG signal channels are translated to a sequence of numeric values, after performing preprocessing (i.e. cleaning, removing noises, and filtering), feature extraction, and selection at the processing stage. Additionally, signal anomalies can be predicted using Deep Learning techniques to avoid or early detect myocardial infarction, arrhythmia, heart failure, etc. over analytics-enabled SCH platforms. Furthermore, real-time and interactive visualizations, insights provision, and recommendations appearing in stakeholders’ mobile devices and web applications are effectively supported by smart visualization platforms and algorithms. For later retrieval, the patient’s ECG data is stored in big data platforms with added security and trusted transaction features enabled by Blockchain to ensure privacy. Other intelligent features including sensor instrumentation for preprocessing data at the edge, device energy harvesting, self-adaptation, and self-learning algorithms accommodating the dynamic nature of the environment support the swift response to emergencies and avoid patient risk of complications [163].

## V. Case Study Analysis on SCH: COVID-19 Pandemic Management

Though COVID-19 is rapidly spreading, most people have experienced only mild to moderate symptoms [164]. Thus far, COVID-19 has claimed many lives, particularly of patients with comorbidities, i.e., the presence of one or more additional health and often co-occurring conditions. COVID-19 has shown the world that it is not fully equipped to confront a pandemic from an effective healthcare perspective. Lockdowns and an indefinite pause on nearly all human activities may not be a long-term solution owing to the devastating economic consequences. In this section, we present a thorough case study on how SCH and the adoption of different technologies have supported the fight against the COVID-19 pandemic, facilitating successful pandemic management and isolation.

### A. Strategies to Combat COVID-19

Different countries are tackling the pandemic differently. In this case study, we focused on the countries that applied emerging technologies in conjunction with SCH for COVID-19 pandemic control and management. We found that using technology coupled with law enforcement helped in flattening the coronavirus curve and saved numerous lives in countries such as China, Korea, Vietnam, and Germany. This is in contrast to other countries, such as Italy and the USA, who have been unable to contain the spread of the COVID-19 pandemic.

**FIGURE 5.**
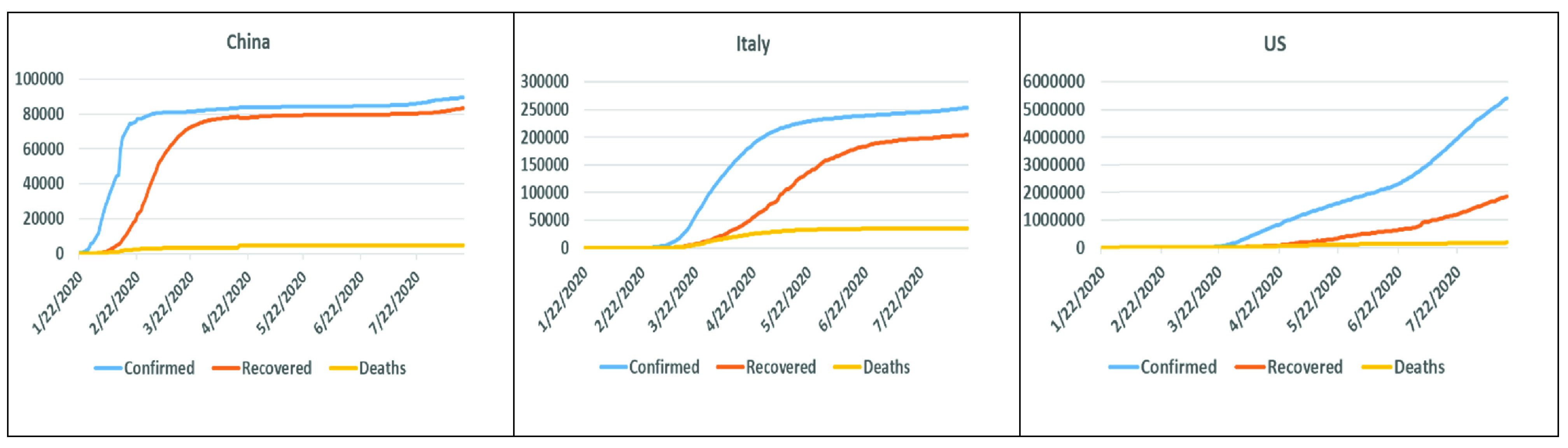
COVID-19 confirmed cases, recoveries, and deaths in China, Italy, and the USA over time.

China reacted to the pandemic early on, whereas Italy was unprepared and underestimated the pandemic; similarly, the USA did not have a clear vision or a consistent strategy to control the pandemic. In this case study, we select three countries that reacted differently toward the pandemic. Fig. 5, shows the coronavirus curves of the confirmed cases, deaths, and recovered cases for the selected countries, namely, China, Italy, and the USA, respectively, according to the statistics recorded between January 22 and August 16, 2020 [165]. It can be observed that the China curves were flattened within a very short time, whereas for Italy, the curves took a longer time to flatten. Moreover, the curves for the USA have not been flattened yet.

**FIGURE 6.**
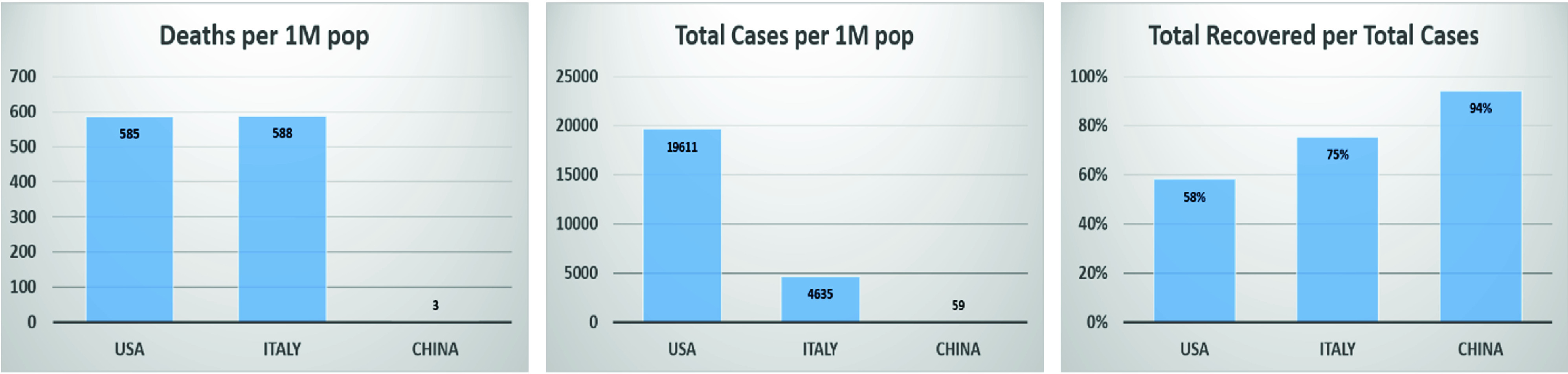
The number of deaths, total cases, and recovered cases, thus showing confirmed cases per million population in all three countries.

Fig. 6, shows the number of deaths, confirmed cases, and the total number of recovered cases per million with respect to the total confirmed cases [166]. Figures 5 and 6 clearly show that the number of deaths and confirmed cases in Italy and the USA significantly exceeded the corresponding numbers in China. However, the percentage of recovery per total number of cases in China surpasses those for both Italy and the USA. China’s success in combating the COVID-19 pandemic can be attributed to several reasons; in the following paragraphs, we explain some of the reasons in detail.

China adopted AI and big data technologies for tracking cases and modeling, efficient diagnoses, patient screening, vaccine development, and 5G enabled patient telemedicine. Alibaba Cloud [167] supports the high computing power required for big data processing analysis. In addition, deep-learning algorithms were applied to the medical knowledge atlas, including high-resolution Computerized Axial Tomography (CAT) scans, to significantly reduce the diagnosis time to a few seconds. Furthermore, China built a new hospital based on an all-on-cloud medical system, which included networking and cloud resource deployment that was completed in only three days [147]. About 22 provinces and cities in China adopted a variety of 5G applications to combat the COVID-19 pandemic [168]. A local telecommunication provider, China Mobile, provided 449 5G-powered infrared thermal imaging temperature measuring devices to 321 schools in one of the provinces. These devices adopt both low-latency 5G and AI algorithms for face tracking and measuring multiple body temperatures at a distance of 10 m for up to 500 people per minute [168]. A study on the extent of utilization of SCH equipment for telemedicine during the pandemic in China showed how the West China Hospital of Sichuan University (WCH) conducts teleconsultations, teleradiology, and tele-intensive care [169]. Other global healthcare networks and providers in general, particularly those in third-world countries, are encouraged to explore the potential of telemedical technology in the fight against COVID-19.

Monitoring and surveillance technologies were well developed and effectively implemented. For example, smart sensor-based glasses were introduced by a Chinese company called Rokid [170]. These glasses are equipped with infrared sensors that can monitor body temperatures of up to 200 people simultaneously by utilization different technologies such as Artificial Intelligence, Augmented Reality, Robotics, and Human-Computer Interaction techniques [171]. Other surveillance technologies such as drones and closed-circuit television (CCTV) cameras were utilized. These cameras are used to monitor quarantined people and thus control the spread of the virus, whereas the drones were used to remind people about social distancing and command them to wear their masks. In contrast, in Hong Kong, quarantined individuals were tracked using a wristband linked to a smartphone app that alerted the authorities in case of violations of the specified zone [172].

China has emphasized the utilization of the existing telecom infrastructure to accommodate the required resources to face COVID-19. Therefore, there has been a 22.61% increase in the daily broadband network traffic, with the infrastructure supporting over a million 4G base stations, 75,000 5G base stations, and 170 million broadband subscribers, and providing a high bandwidth backbone to fulfill the requirements of the highest levels of data consumption [147].

With an increased demand for teleworking and distance learning, a myriad of multifaceted remote applications has emerged to support different industries, with over 700,000 users [147]. In addition, existing and newly created social media tools and smart applications were adopted to track people’s mobility in public transportation systems [173]. Furthermore, robots were used at Wuhan Thunder Mountain Hospital to perform multiple tasks, including disinfecting hospital buildings, distributing hospital supplies, and performing temperature checkups for patients. Thus, they helped reduce the interactions of healthcare workers with patients, thus drastically reducing the transmission of COVID-19 between people and the number of medical task force needed [147]. These medical robots adopt a myriad of technologies to perform the required tasks such as cleaning, sterilization, carrying and moving objects, nursing, and others. For instance, in order to control navigation, adaptive embedded controllers are adopted to satisfy complexity and agile requirement. Moreover, wireless power transfer and several renewable energy sources are adopted to guarantee continuous power charge.

Unfortunately, authorities in countries such as the USA and Italy did not take the necessary measures needed to control the spread of the pandemic. Italy suffered numerous deaths that by the end of March 2020, constituted Italy’s highest number of casualties since World War II. Many reasons contributed to the loss of control over the spread of COVID-19 in Italy: the lack of data arising from unsystematic data collection in some hospitals and the lack of coordination between healthcare systems in public and private sectors, testing centers, and physicians [177]. Towards the end of March 2020, in Lombardy alone, the number of cases exceeded 41,000 and the deaths exceeded 6,300, which represent 42% and 59%, respectively, of the total cases in Italy [174].

**FIGURE 7.**
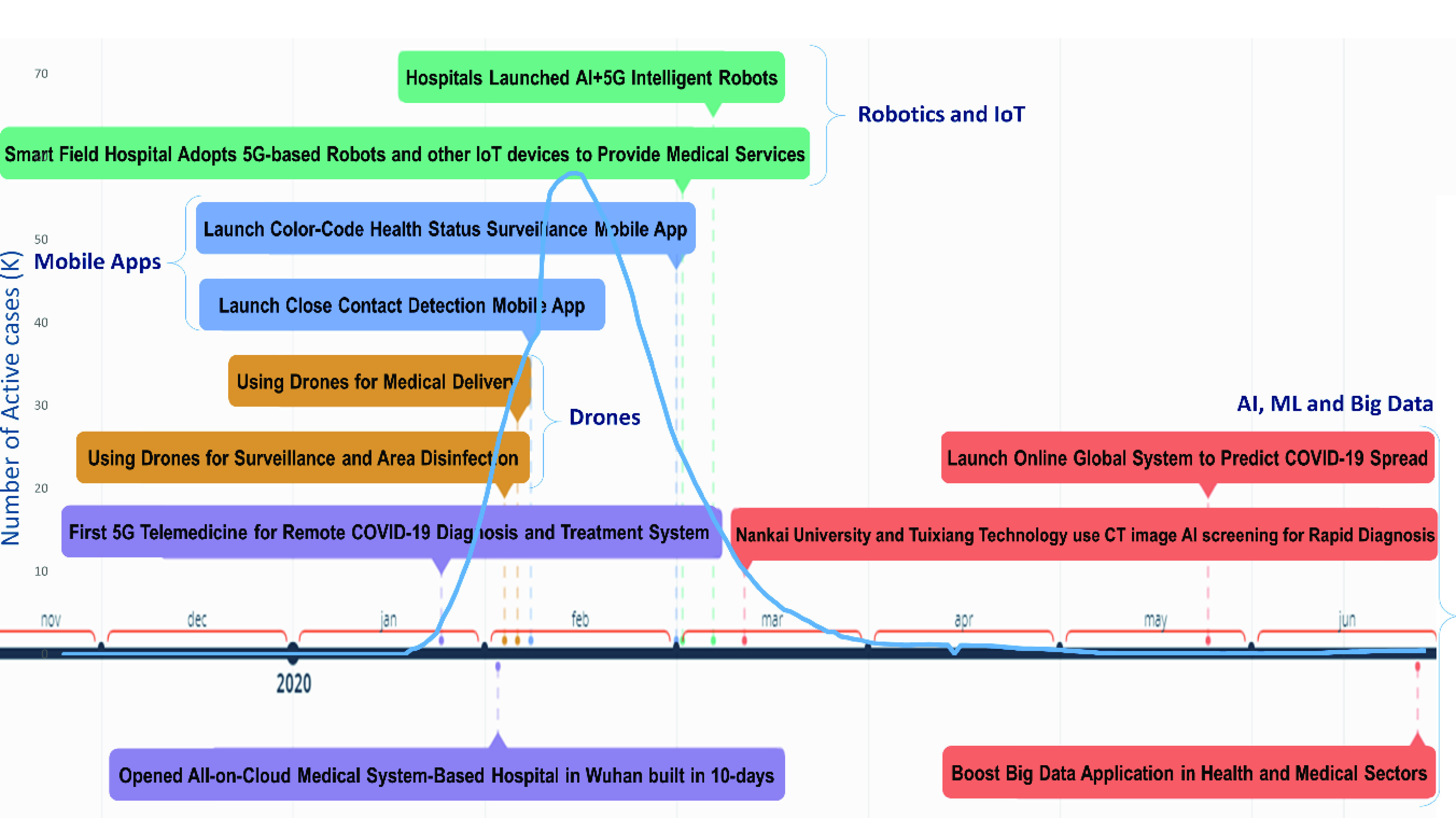
Timeline of China’s SCH adoption to help flatten the curve of new active COVID-19 cases.

China has been an exemplary model in terms of epidemic readiness and control. Its fast-paced tactics, innovation, and adoption of state-of-the-art technologies marked its success in the fight against the coronavirus [175]. Fig. 7, shows the timeline of the SCH implementation for COVID-19 pandemic management in China. The curve shows how swiftly and radically the use of technologies, including AI, big data, robotics, IoT, drones, and mobile applications, has reduced the number of new cases causing the curve to flatten in less than two months.

### B. Technologies Used to Combat COVID-19

AI, Big Data, IoT, Smart mobile applications, and Drones have been extensively used during the different phases of combating the pandemic including lockdown, movement tracking of the people, maintaining social distancing, and reducing healthcare workers’ proximity to patients. The following subsections provide different examples of technology-based applications as adopted by different countries in the fight against the pandemic.

#### 1) Monitoring and Tracking Applications

Smartphone apps provide tremendous opportunities to monitor the spread of COVID-19-related rumors, raise public awareness, enable mobile tracking, and enhance the delivery of preventive actions and clinical care [176]. Massachusetts Institute of Technology researchers have stressed on using the geographic information system (GIS) with IoT mobile data to help detect infections and identify and track persons who may have come in contact with infected patients [177]. For example, connected temperature sensors produce real-time patient data which is relayed through IoT Access Controller and wirelessly transmitted to a medical worker station to allow continuous monitoring. This gateway has the capability of allowing multiple simultaneous Bluetooth Low Energy devices enabled with long-range connectivity to provide several rooms coverage in the hospital. Furthermore, data was collected from over a million connected thermometers to generate maps that help to locate high population with fever. Other IoT devices include IoT buttons, which connect automatically to the LTE-M network and are used to send direct alerts to management regarding cleaning or maintenance issues. The latter are positioned in different locations such as patient rooms, restrooms, and common areas. The E-quarantine [178] system tracks the flow of patients and predicts the number of emergency cases within 24 h by relying on the level of blood pH, heart rate, blood pressure, body temperature, and rate of breathing recorded in its database.

Singapore and South Korea combined different types of information such as location data, video, and credit card transaction information for tracking the virus. Singapore adopted the ***TraceTogether*** [179] application that depends on Bluetooth signals exchanged between cell phones to track people who came into contact with infected people [172]. Additionally, South Korea built a smart quarantine information system and implemented a self-health check mobile app. These applications combine mobile phone location data, credit-card transaction records, and video surveillance for tracking people who may have come into contact with individuals specified on a list of infected patients [180].

Different mobile apps were developed in India to respond to the pandemic. The government used phone call records, video surveillance, and cell location data for tracking the individuals who came into contact with coronavirus patients [172]. A smartphone application ***Aarogya Setu*** [181], provides different digital services to facilitate contact tracing, syndromic mapping, and self-assessment. This app received more than 100 million downloads in the first 40 days of its launch [182]. Another app called ***COVID-19 Feedback*** [183] helped the government to collect information about patients’ health records, thereby assisting in pinpointing the worst-affected areas and improving the testing facilities. Moreover, the ***Quarantine Monitor Tamil Nadu*** [184] app was developed to monitor people under home quarantine [185].

The Ministry of Health in Vietnam adopted the ***NCOVI*** [186] app, which provides a map of the location of infected cases and areas and reports new cases and self-health status. It also shows the real-time movement of quarantined patients. Although it involves many privacy violations, the app was ranked fourth in terms of downloads among free health and fitness apps in Vietnam’s iOS app store. It appears that in the face of the fear of COVID-19, people prefer to lower their firewalls and sacrifice their privacy. This is an interesting and simultaneously concerning insight that calls for further research. Another Bluetooth-enabled application named ***Bluezone*** [186] provides notifications to users when they approach infected persons [187].

Germany promoted social distancing using information systems and technology, a strategy that yielded good results in controlling the virus. This is achieved through continuous testing and tracking of people. Different apps were developed for this purpose, such as, the ***Corona-Datenspende*** (Corona Data Donation) [188] smartwatch app, which analyzed activity logs and heart rate information along with address information to detect potential COVID-19 symptoms. In addition, contact-tracing apps alert persons who come in close contact with infected patients. This is achieved through centralized systems at the government side or decentralized ones based on Bluetooth connections between individuals’ mobile phones. Owing to this strategy, the number of infected people aged above 70 years (19%) was considerably lower than that in Spain (36%) and Italy (39%). The death cases constituted 4.6% of the overall cases as of May 2020, which markedly lower when compared with 14.1% and 12% death cases recorded in Italy and Spain, respectively [189].

Nevertheless, the government in the USA is still investigating the possibility of using the location information collected from Facebook, Google, and other vendors to track the virus; however, they are still facing several privacy issues [172].

Some other mobile phone apps developed worldwide are as follows: ***nCapp*** [190], ***Stop Corona*** [191], ***Social Monitoring*** in Russia [192]; ***Civitas*** in Latin America [193]; ***StayHomeSafe*** in Hong Kong [194]; ***Coalition*** in the USA [195]; and ***BeAware Bahrain*** [196].

#### 2) AI-Based Diagnosis Applications

AI-based applications contribute to accelerating the diagnosis process of COVID-19, analytics, and predictions of the spread of the pandemic. For example, South Korea managed to contain coronavirus using AI-based diagnosis testing kits in less than a week. They developed a Chest X-Ray AI Image Support Decision Tool that identifies patients requiring immediate care within 3 s based on abnormalities in the lung X-ray images. Further, they launched an all-in-one medical platform named AiHub, which applies technologies based on AI and big data for the diagnosis of different diseases. The government also provided AI-based chat and voice robots that advise the public on ways to respond to the virus or call for help. The authorities are working on transforming affected cities such as Daegu into a smart city by 2021 to control a possible future wave of the pandemic [180].

Taiwan integrated its health insurance database with the immigration department using big data for analytics to assist in the diagnosis and generate real-time warnings based on travel information and medical symptoms [197].

#### 3) Drones

IoT-based drones can be used for different purposes, including surveillance, mass testing, announcement, diagnosis, delivery, and disinfection of areas [198]. Diagnosis drones such as thermal-imaging drones can be used for monitoring and detecting infected cases by checking the temperature, vital signs such as heart rate, and symptoms such as sneezing or coughing remotely [199], [200]. China and India applied surveillance drones for crowd control [171]. Delivery drones were deployed in Ghana, China, and Canada for delivering swab test kits [201]–[203]. They can also be used to deliver other goods and groceries to avoid visits to supermarkets and reduce potential human physical interactions. An example of a disinfectant drone was that developed by DJI [208], a Chinese company; this drone can disinfect one hundred square meters in one hour. These drones were used in Spain as well [171]. Multipurpose drones called Corona Combat with similar functionalities were used in China.

#### 4) Robotics

Humanoid robots, autonomous vehicles, and other intelligent robots can help reduce physical interactions between healthcare workers and infected persons [204]. Robots can help in many tasks such as preparing food, providing medication to patients, and cleaning and disinfecting health facilities. For example, Asimov Robotics in India provided services to help quarantined patients [171]. In Singapore, the *eXtremeDisinfection* robot (XDBOT) equipped with wireless control and a mobile platform was used to disinfect contaminated areas, including the area under the patients’ beds [171]. Boston Dynamics’ quadruped robot, Dr. Spot, is being used as a telemedicine robot at a Boston hospital. Medical personnel can remotely videoconference with patients using an iPad and the two-way radio on Spot’s back [205]. Several nurse robots, such as Paro (AIST, Japan), Pepper (Softbank Robotics, France), and Dinsow (CT Asia Robotics, Thailand) are being used to help elderly patients in both lifting tasks and clinical assistance [206]. Companies are reopening in the pandemic, with robotic security guards, where humanoid robots such as Pepper are used; these robots have extended features of AI-enabled mask detection and Thermovision subsystem (2020-03 Coronavirus update) [207], allowing them to acquire the customers’ body temperature.

## VI. Challenges and Future Directions of SCH

There are several modules, processes, a multitude of technologies, variable application contexts, and diverse stakeholders involved in SCH systems that reveal a variety of challenges. In this section, we will address a couple of the relevant challenges in SCH, accompanied by future directions.

### A. Challenges of SCH

Following are certain challenges that any potential SCH infrastructure must resolve in order to effectively function.

#### 1) Security and Privacy

IoT-enabled SCH poses major challenges to patient privacy. In SCH applications, a massive amount of data are continuously collected from and about patients. These data can be penetrated, copied, or deformed by unauthorized parties through malicious attacks such as tag cloning, spoofing, radio frequency (RF) jamming, and cloud polling. RFID tags that are not password-protected or protected by over-the-air (OTA) encryption can be cloned or replicated. An RF jammer is a device intended to prevent the reception of radio transmissions by a receiver relevant to its function. This type of attack can interrupt the functionalities of life-monitoring systems, sometimes causing loss of life. In cloud polling, traffic is redirected, allowing unauthorized command infusions directly into a device through a man-in-the-middle attack.

In addition, denial of service (DoS) attacks can interrupt healthcare systems, consequently risking people’s lives. A common solution to DoS is redundancy; however, in a healthcare environment, resource duplication may not always be feasible. Many healthcare centers consider life-critical monitoring devices as extremely important to purchase and install. Hence, the prompt discovery of potential security threats remains a persisting challenge; with increased connectivity, the security threats are worsening. In SCH, wearable devices are used to accomplish critical health goals. However, the unavailability of security standards for these devices in conjunction with the ease of use of powerful search engines such as Shodan [208], which enables searching Internet-connected devices, exposes these wearable devices to various security attacks.

To integrate voice-activated assistants into healthcare, the design of voice-enabled user interfaces must consider the different needs of the end-users and their settings (e.g., background noise and multiple users -the patient, their caregiver, attending nurse, etc.- of a single device in a home). However, the unique privacy and security issues that arise with the use of the devices that are always listening in the background [16] is a risk. Many potential concerns exist regarding protection and privacy when using voice-activated assistants. The privacy consequences of using these devices may not be understood or known by customers.

To help tackle the spread of COVID-19, a wide range of surveillance technologies have been rapidly developed and deployed. This action has been justified by the argument that they are vital to suppressing the virus. In this case, civil liberties have been sacrificed for public health [209]. Privacy, confidentiality, and security are major concerns to both patients as well as physicians; hence, implementing security standards and defense mechanisms is imperative.

#### 2) Connectivity and Heterogeneity of Connected Devices

A major challenge to SCH is connectivity. Many devices need to connect and continuously share data. Sensors of different types collect data and communicate with servers in their own language. Each manufacturer has their own communication protocol. Therefore, sensors manufactured by different manufacturers may not be able to communicate with each other. This divergence in communication protocols and software environments results in major challenges when designing large-scale SCH. Communication interfaces, data conversion and transformation, and protocol translation/conversion become a must, thus adding more technical complexities to the implementation of SCH.

Furthermore, this enormously connected smart health system should allow patients and medical staff to move freely across several physical locations, which may be covered by different wireless networks, without interruption. However, mobility can result in collisions, particularly when several wireless networks operate within the near range. Collision causes many disruptions. It reduces the performance and may result in catastrophic situations, especially with regard to healthcare delivery. Consequently, ensuring that medical devices accurately operate when connected using different types of wireless technologies is crucial.

Interoperability is a major requirement for efficient and successful SCH. A truly interoperable SCH system is one in which data can flow in one-to-one and one-to-many connections, generating the exchange of valuable data among multiple interfaces. This accentuates the need for devices that are compatible with various transmission formats and protocols.

#### 3) Usability and Accessibility

SCH systems utilize advanced technology that may not always be easy to use by physicians or patients. The presence of many features and configurations can, at times, make a system too complex, which subsequently discourages people from learning how to use it. Proper training and careful design of these devices are essential to ensure ease and continuity of use.

#### 4) Big E-Health Data Analytics Challenges

The challenges of big data healthcare analytics are classified into data and process-related, manpower-related, domain-related, and organizational challenges [66]. Significant multidimensional data handling, including image diagnostics, generate tremendous volumes of information; hence, scalability of storage is required [70]. The overall quality of health data is influenced by the size, speed, and formats of the generated and processed data. Decision-making insights rely on the quality of big health data. Therefore, data quality should be preserved at all stages of big data processing [210], in particular, during the preprocessing stage that involves sub-processes such as cleaning, integration, filtering, and normalization. GIS and big data technology have played an important role in combating COVID-19. Seeking techniques to adapt conventional technological approaches, data aggregation, knowledge discovery, and increased speed and precision are the prevalent challenges [211]. The use of GIS in pandemic tracking poses challenges in terms of data collection from heterogeneous data sources. These challenges are forcing governments, companies, and academic institutions to collectively facilitate policy formulation. Spatial analysis strategies for big data, including multiscale dynamic mapping for epidemics, are research areas that need further research.

#### 5) Remote Monitoring, Mobility, and Other Challenges

The introduction of IoT and mobile applications presents a range of challenges in terms of remote monitoring, connectivity capabilities, the nature of smart computing systems, and the provision of context-aware facilities. In IoT sensor networks, parameters such as the number of sensors are selected based on the application and user-specific needs [129]. Although considerable progress has been made in terms of sensors and IoT, no devices that match the accuracy of hospital-grade devices without compromising either energy efficiency or wearability are available [110]. Energy consumption, battery life, and design of miniaturized energy sources still present major challenges for mobile devices and devices in sensor networks. At large scales, scalability, maintaining around-the-clock connectivity, real-time monitoring, compression, transmission, and on-the-fly health data processing are serious challenges.

The need for cooperation among multiple robots that share spaces with humans represents a key issue in smart robotic environments [26]. In human-robot interactions, smart robots are supposed to respond to well-established human gestures. Such interactions have been studied in recent years, and constraints related to eye-tracking, voice interaction, and biological recognition are being tackled. For adopting drone-based technology, the need for trained personnel and the lack of facilities such as runways pose potential issues. Drones cannot carry heavy payloads or carry goods over long distances [69]. In most nations, global regulations have previously restricted the use of drones to military uses because they are likely to be lost, destroyed, or physically hijacked during open-air operation [212].

In predictive and personalized medicine, many AI-based deep learning models do not handle data of poor quality, and building a clinically useful prediction model is a challenge. At the personalized level, a predictive model should be continually modified, well-tuned, and delivered with ample time for early and successful clinician intervention. Owing to irregular or missing data and the variability of patient comorbidities and treatment, modeling the continuous existence of chronic diseases and development over time is a challenge [213]. In addition, smart AI-enabled devices may provide insights or decisions on the patient’s diagnosis, disease predictions, and/or warnings. In several situations, a decision based on AI, using DL or ML, may be incorrect. In the event of a false statement or misdiagnosis, an ethical dilemma regarding fixing the responsibility may arise. We are optimistic that SCH will be broadly adopted in the immediate future if the majority of the challenges are adequately addressed.

### B. Future-Generation SCH Solutions and Systems

This work evidently shows that the SCH system leverages new technologies to provide an efficient, cost-aware, fully connected, and powerful monitoring system. Considering the increasing network traffic, the following recommendations regarding capabilities of future traffic networks are inferred: incorporation of self-configuration, self-optimization, self-healing, and self-protection techniques [102]. Portable equipment makes imagery easily available to patients, particularly in rural areas, and 3D printing, combined with imaging research, increases patient awareness and allows physicians to view complex anatomy before surgery [23]. Using 3D printing, custom implants can be created to fix a host of anomalies in the bones and can be used in craniofacial surgery, the study of human anatomy, and tissue engineering among other applications [214]. The knowledge can be input into robotic surgical systems to conduct procedures with a higher degree of accuracy, resulting in optimal success [48]. In the near future, three-quarters of Internet users may only be able to access the Internet through smartphones. This indicates the opportunity to use mobile apps to track healthcare, and thus, mHealth holds tremendous untapped potential, particularly in the field of personalized healthcare [215].

Microblogging sites like Twitter and social media apps [216], [217] have been used to identify activities, feelings, and other critical information from online social interactions in greater depth and at a faster rate. Mental wellbeing is a part of one’s physical wellbeing and cognitive conduct, and telehealth and teleconsultation are valuable means to ensure regular patient engagement and mental health services during this time of social distancing and quarantine [218]. Isolation and loneliness are also on the rise, leading to the so-called “loneliness syndrome”, endangering wellbeing across ages worldwide. It has been aggravated with global social-distancing interventions since the COVID-19 crisis, and this situation suggests the need for companion robots to alleviate feelings of seclusion [219].

We expect that SCH will evolve and innovate to provide efficient, flexible, and cost-aware solutions, applications, and services to patients, healthcare practitioners, organizations, and even governments; this will enable the implementation of next-generation healthcare in the future. Its adoption by many countries will change the way of providing health services, monitoring patients, and managing diseases and pandemics.

## VII. Conclusion

SCH is an emerging research area that has attracted the interest of researchers, industries, and governments worldwide because of its potential to transform the healthcare sector into an efficient ecosystem. Herein, we explored this subject and proposed a comprehensive classification of the existing work on SCH solutions. This classification considered the main SCH-enabling technologies, the application contexts for which SCH is adopted, and the futuristic developments of SCH. We also proposed an architecture that pertains to the technological aspect of SCH solutions involving the environment in which SCH is deployed, along with the key stakeholders.

The research also provided a bird’s eye view of the current COVID-19 pandemic in light of the developed SCH architecture and classifications. This will provide insights and guidelines to different stakeholders involved in the fight against the pandemic to develop more effective technologies and techniques. The processes and framework outlined herein describe details along with a case study on the strategies adopted by different countries in combating the pandemic. This further confirms the global reach of the problem and the need to join forces on a global scale to successfully control the pandemic using SCH. The work also attempted to address the present global crisis and endeavored to provide possible technological solutions and options. SCH clearly represents an effective solution in the fight against this catastrophic pandemic, which has thus far caused death and havoc everywhere, crippling the economies of several countries. Through the COVID-19 Pandemic Management case study supported with evidenced data, we proved that technology has helped in delimiting and decreasing the spread and facilitated in China’s comeback, which is not the case with other countries we have compared to like Italy and the USA. This research clarifies that the road to the full resolution of this pandemic is fraught with many challenges. One of the greatest concerns baffling health experts is related to the deaths of asymptomatic COVID-19 virus carriers. Data can shed more light on the causes of such deaths, for example, by revealing tacit patterns, and SCH, which is proficient in collecting and analyzing such data can play a decisive role in reducing and even preventing such deaths.

It appears that COVID is here to stay for a while, and lockdowns and staying at home are not long-term solutions. However, living with the virus may likely be the new norm in human lives, and hence, the future seems to be a connected world with a smartly connected healthcare system. The pandemic has markedly accelerated the efforts toward building an SCH and has pushed many nations to re-prioritize their resources to infuse more ubiquitous smartness in their operations in general and in healthcare to combat COVID-19. SCH, with the advantages of improved quality of care and accessibility for all, will help transform reactive healthcare into proactive healthcare.

The power of data obtained from multiple medical equipments, mobile devices, miniature sensors, and other sources can be harnessed by SCH. In addition, AI can provide insights, customize health experiences, and suggest lifestyle and wellbeing improvements to users at the cloud or edge, thus empowering them. To this end, our proposed architectural model of the SCH and the literature classification of the state of the art technologies and solutions provided insights to researchers and healthcare organizations to implement and deploy SCH to combat pandemic issues.

Yet, there persist several challenges related to SCH adoption and the realization of the full potential of such solutions. The COVID-19 pandemic is one of the greatest medical and social challenges in decades. The pandemic has exposed the immediate need for a total transition to a modern healthcare paradigm. For emergency cases, SCH may no longer be considered an alternative or a complementary solution. It can be considered as a routine for patients with recurrent comorbidities for which treatment assures the appropriate quality of care. Now that the world is moving toward digital healthcare, emerging technologies such as IoT, AI, and telemedicine should be incorporated into international and national public health planning guidance. Improving the technical expertise of healthcare stakeholders, public training, and health data exchanges while fulfilling privacy and security requirements are the key challenges in SCH adoption.
